# Immunohistochemical Expression Levels of Epidermal Growth Factor Receptor, Cyclooxygenase-2, and Ki-67 in Canine Cutaneous Squamous Cell Carcinomas

**DOI:** 10.3390/cimb46050297

**Published:** 2024-05-19

**Authors:** João Miguel Luís, Rita Files, Cláudia Cardoso, José Pimenta, Gabriela Maia, Filipe Silva, Felisbina L. Queiroga, Justina Prada, Isabel Pires

**Affiliations:** 1 Department of Veterinary Sciences, University of Trás-os-Montes and Alto Douro, 5000-801 Vila Real, Portugal; joaomiguelluis1992@gmail.com (J.M.L.); ritafiles2000@gmail.com (R.F.); gabriela.maia98@gmail.com (G.M.); jprada@utad.pt (J.P.); 2 Animal and Veterinary Research Centre (CECAV) and Associate Laboratory for Animal and Veterinary Sciences (AL4AnimalS), University of Trás-os-Montes and Alto Douro, 5000-801 Vila Real, Portugal; josepimenta@utad.pt; 3 CIVG—Vasco da Gama Research Center/EUVG, Vasco da Gama University School, 3020-210 Coimbra, Portugal; 4 Centre for the Study of Animal Science, CECA-ICETA, University of Porto, 4099-002 Porto, Portugal

**Keywords:** EGFR, Ki-67, Cox-2, canine, squamous cell carcinoma, aggressiveness

## Abstract

Squamous cell carcinoma (SCC) stands as the second most prevalent skin cancer in dogs, primarily attributed to UV radiation exposure. Affected areas typically include regions with sparse hair and pale or depigmented skin. The significance of spontaneous canine cutaneous SCC as a model for its human counterpart is underscored by its resemblance. This study assesses the expression of key markers—Epidermal Growth Factor Receptor (EGFR), Cyclooxygenase-2 (Cox-2), and Ki-67—in canine cutaneous SCC. Our objective is to investigate the association between their expression levels and classical clinicopathological parameters, unraveling the intricate relationships among these molecular markers. In our retrospective analysis of 37 cases, EGFR overexpression manifested in 43.2% of cases, while Cox-2 exhibited overexpression in 97.3%. The EGFR, Cox-2 overexpression, and Ki-67 proliferation indices, estimated through immunohistochemistry, displayed a significant association with the histological grade, but only EGFR labeling is associated with the presence of lymphovascular emboli. The Ki-67 labeling index expression exhibited an association with EGFR and Cox-2. These findings propose that EGFR, Cox-2, and Ki-67 hold promise as valuable markers in canine SCC. EGFR, Cox-2, and Ki-67 may serve as indicators of disease progression, offering insights into the malignancy of a lesion. The implications extend to the potential therapeutic targeting of EGFR and Cox-2 in managing canine SCC. Further exploration of these insights is warranted due to their translational relevance and the development of targeted interventions in the context of canine SCC.

## 1. Introduction

Squamous cell carcinoma (SCC) encompasses a variety of tumors originating in various anatomical locations, characterized by shared genetic mutations and expressions of squamous differentiation markers. Among the multiple types of SCC, cutaneous squamous cell carcinoma (cSCC), an invasive and metastatic form originating from keratinocytes, is the second most common cutaneous neoplasm diagnosed in dogs [1,2,3,4,5,6,7].

Canine cSCC is associated with factors such as chronic sun exposure, absence of pigment in the epidermis, and sparse fur at the tumor site. However, its occurrence is not restricted to sun-exposed areas [8,9]. Canine cSCCs usually show local invasion, resulting in the gradual loss of underlying tissues. Although metastasis is considered rare or late in the progression of the disease, specific subtypes, such as nail bed cSCC, exhibit more aggressive behavior [10].

In the human context, cSCC is a public health challenge, due to its high incidence and associated medical costs. Risk factors include genetic predisposition, family history, pigmentation, exposure to ultraviolet radiation (UVR), human papillomavirus (HPV) infection, immunosuppression, and smoking [11,12,13,14]. cSCC has been classified into three subtypes—well-differentiated, moderately differentiated and poorly differentiated tumors—with well-differentiated being the most common, characterized by the presence of ‘keratin pearls’ (concentric layers of squamous cells with varying degrees of keratinization towards the center of the tumor nests) [9]. Several molecular pathways are implicated in the development of cSCC, including mutations in the P53 gene, alterations in suppressor genes such as CDKN2A and NOTCH, oncogenes such as RAS, and activation of the NF-kB, MAPK, and PI3K/AKT/mTOR pathways. In addition to genetic mutations, epigenetic changes can also play a role in this process [15,16,17,18,19].

Tumor biomarkers play a crucial role in oncology, offering a less invasive and low-cost approach than traditional methods, such as imaging and histology. In humans, these biomarkers provide information on the presence of tumor tissue, disease progression, prognosis, and detection of recurrences. However, in veterinary medicine, the use of biomarkers is still limited, due to their low sensitivity and specificity. The high rate of cell proliferation in malignant tumor tissues highlights the importance of molecules that are increasingly expressed as biomarkers [20,21,22,23]. These biomarkers, in oncology, can be grouped into diagnostic, prognostic, treatment, and prevention, identifying key mutations, molecular pathways, and other markers that guide individual therapy and estimate different outcome risks. Despite the challenges in veterinary medicine, research continues to improve the efficacy of these tools, aiming for benefits similar to those achieved in human oncology [20,21,22,23,24].

In normal skin tissue, the Epidermal Growth Factor Receptor (EGFR) plays a vital role in cell proliferation, differentiation, and survival, as well as in the migration of keratinocytes during the healing of skin lesions [25,26]. However, its deregulation is a significant driver in cancer development, transforming its ligands and establishing an autocrine signaling cycle [27,28,29]. The EGFR family of related transmembrane tyrosine kinase receptors, when activated by ligands such as EGF or TGF, triggers signaling pathways that affect essential cellular processes, such as cell division, growth, differentiation, metabolism, and apoptosis. Dysregulation of EGFR is observed in several types of tumors [30,31,32,33].

Cox-2, an enzyme that converts arachidonic acid into prostaglandins, participates specifically in inflammatory processes [34,35]. Cyclooxygenase-2 (Cox-2) is frequently expressed in various types of cancer, playing multifaceted roles in carcinogenesis and resistance to therapies. Its presence in the tumor microenvironment contributes to cancer stem cell-like activities, promoting apoptotic resistance, angiogenesis, inflammation, invasion, and metastasis [36,37]. Cox-2 is secreted by cancer-associated fibroblasts, type 2 macrophage cells (M2), and cancer cells in the tumor microenvironment. Its presence induces cancer stem cell-like activities, promoting apoptotic resistance, proliferation, angiogenesis, inflammation, invasion, and metastasis of cancer cells. Cox-2-mediated hypoxia in the tumor microenvironment and positive interactions with YAP1 and anti-apoptotic mediators contribute to cancer cells’ resistance to chemotherapeutic agents [37,38,39].

The Ki-67 protein, present in the nuclear DNA of all vertebrates, is commonly used as a proliferation marker in tumors [40]. During the cell cycle, its location varies, found in the peri nucleolar region in the G1 phase, in the dense fibrillar component of the nucleolus during interphase, and associated with chromatin during mitosis [41,42]. The regulation of Ki-67 levels is complex, with maximum expression in mitosis and minimum expression at the end of G1 [43]. After passing the G1 restriction point, CDK4/CDK6 activation triggers RB phosphorylation, increasing Ki-67 mRNA transcription. The ubiquitin–proteosome system mediates the subsequent degradation of the Ki-67 protein in late mitosis and early G1, highlighting the complexity of cell proliferation regulation [42,44,45]. 

Due to their established roles in the aggressiveness and prognostication of several canine tumors, and the availability of therapeutic options regarding these markers for dogs, the main objective of this research is to analyze the expression levels of EGFR, Ki-67, and Cox-2 in canine cutaneous squamous cell carcinomas. The approach includes immunohistochemical methods through which to evaluate the expression levels of these markers and subsequently investigate the possible correlation between them and the clinicopathological characteristics of the tumors. In addition, this research aims to explore the interrelationships between the markers under study.

## 2. Materials and Methods

### 2.1. Tissue Samples

This study analyzed 37 cases of cutaneous squamous cell carcinomas in dogs. The tumor samples were obtained from the Histology and Pathology Laboratory of the University of Trás-os-Montes and the Alto Douro archive. These tumors were collected during surgical procedures or necropsies, previously fixed in 10% buffered formalin, and embedded in paraffin. Clinical data, such as age, gender, and breed, were recorded for each animal.

The most common breeds of the animals with SCC were Boxers (*n* = 8) and Indeterminate (*n* = 4). Regarding sex distribution, 20 were females and 17 were males. The animals’ ages ranged from 1 to 17 years, with an average age of 8.5 years (±2.60).

Microscopic analysis involved staining 3 μm thick sections using hematoxylin and eosin. Two pathologists (IP and JP) conducted independent analyses for each sample, examining all slides in each section in detail. The histopathological diagnosis followed the World Health Organization (WHO) classification of animal tumors. A Nikon Eclipse E600 microscope, with a Nikon DXM1200 digital camera (Nikon Instruments Inc., Melville, NY, USA), was used for microscopical observations and image capture.

### 2.2. Histopathology

The following parameters were evaluated: keratinization/differentiation, nuclear pleomorphism, mitotic count, invasion pattern, invasion stage, and lymphoplasmacytic infiltration. Regarding the assessment of the mitotic index, cells in the process of mitosis were counted in 10 high-magnification fields using a 40× objective. These parameters were used to classify the histological degree of malignancy into three categories, following the methodology proposed by Annertoth et al. (1984) [46]. As described in Table 1, the cumulative scores of each parameter defined the histological grade of malignancy, as follows: Grade I, characterized by scores ranging from 5 to 10, indicates well-differentiated tumors. Grade II, with scores between 11 and 15, signifies moderately differentiated tumors. Lastly, Grade III, whose scores exceed 16, represents poorly differentiated tumors. Emboli, ulceration, and necrosis were also recorded [46].

### 2.3. Immunohistochemistry

For immunohistochemical analysis, 3 µm thick sections were used. The primary antibodies used were Ki-67 (Ki-67, clone MIB I, Dako^®^; dilution 1:50; 24 h at 4 °C), Cox-2 (Clone SP21, Transduction Laboratories ^®^, Lexington, KY, USA; dilution 1:40; 24 h at 4 °C) and EGFR (clone 31G7, Invitrogen ^®^, Paisley, Scotland, UK; 1:100 dilution; 45 min at room temperature). 

Visualization of primary antibodies was achieved using the NovolinkTM Polymer Detection System (Leica Biosystems ^®^, Newcastle, United Kingdom), with 3,3′-diaminobenzidine tetrachloride (DAB) as a chromogen, following the manufacturer’s instructions. In summary, the sample processing began with a 15 min dewaxing phase. This was followed by a hydration process using a graded series of alcohol (100%, 95%, 80%, and 70%), with each stage taking 5 min. For antigen retrieval, the samples were immersed in a citrate buffer solution (10 mM, pH 6.0 ± 0.2) and heated in a 750 W microwave for three cycles of 5 min each. A 3% hydrogen peroxide solution was used for 30 min to suppress peroxidase activity. A 5 min application of a Protein Block followed. The samples were then incubated overnight at 4 °C with the MAC387 antibody (AbDSerotec, MorphoSys UK Ltd., Kidlington, Oxford, UK; Clone MCA 874G) at a dilution of 1:100 in PBS. Subsequently, a post-primary reagent and Novolink Polymer were each applied for 30 min. After rinsing with PBS, the samples were treated with 3,3-diaminobenzidine (DAB) for 10 min for development and counterstained with Gill’s hematoxylin for one minute. The sections were then washed and dehydrated using alcohol (95%, 95%, 100%, and 100%) for 3 min at each step, cleared in xylene, and mounted with Entellan mounting medium (Merck^®^). In previous works, all antibodies showed cross-reactivity in canine tissue samples [47,48,49,50].

Negative controls were established by excluding the primary antibody. For positive controls, various samples were employed, as follows: newborn dog kidney tissues were used to validate Cox-2 staining; normal skin and breast tumor samples were utilized for EGFR; the basal layer of normal skin was used for Ki-67.

Each sample underwent independent evaluation by two observers (IP and JP), both unaware of the clinical and pathological details. For this analysis, they used a Nikon Eclipse E600 microscope equipped with a Nikon DXM1200 digital camera, supplied by Nikon Instruments Inc., Melville, NY, USA. In instances of discrepancy, a third reviewer (LD) was consulted for further assessment. Final scoring was established through a consensus discussion among the reviewers.

### 2.4. Quantification of Immunostaining

#### 2.4.1. Quantification of EGFR Immunostaining 

EGFR antibody expression was evaluated using a semiquantitative method, and immunoreactivity was considered when a brownish marking was observed on the cytoplasmic membrane of the cells. The intensity of membrane labeling was evaluated as either weak (1), moderate (2), or strong (3). Overexpression was considered in cases where the membrane marking had strong intensity [51].

#### 2.4.2. Quantification of Proliferation Index (Ki-67 Immunostaining) 

The evaluation of Ki-67 expression was conducted using a quantitative method. Immunoreactivity was considered when labeling occurred in the nucleus, regardless of the intensity [52]. Detailed observation of each preparation was conducted, choosing the tumor area with the highest nuclear positivity and staining homogeneity. Each selected area was analyzed using a 40× objective, and the fraction of positive nuclei was determined in terms of percentage, accounting for at least 1000 tumor cells in 8 to 10 fields. The same observer performed all counts using a Nikon FXA^®^ microscope with a checkerboard eyepiece, considering cell morphology [52]. Subsequently, tumors were classified into two groups (low and high), using the mean Ki-67 positivity values as the cutoff.

#### 2.4.3. Quantification of Cox-2 Immunostaining

The evaluation of Cox-2 immunolabeling employed a semi-quantitative approach, according to the method described in [53]. This approach considers the proportion of positively stained tumor cells and their staining intensity. The percentage of positive cells was assigned scores from 1 to 4, with 1 indicating ≤10% positivity, 2 indicating 11–50%, 3 indicating 51–80%, and 4 indicating >80%. Similarly, staining intensity was rated on a scale of 1 to 3, corresponding to weak, moderate, and strong staining, respectively. The final score, used to classify the samples, was derived by multiplying the scores for extension and intensity. The mean IHS value established the cutoff point (HIS = 6). Samples were then categorized as having low expression (score ≤ 6) or high expression (score 7–12) [54]. 

### 2.5. Statistical Evaluation

To verify whether the Cox-2, Ki-67, and EGFR markers were statistically related to the histopathological parameters (histological grade and emboli) to be evaluated, or to each other, the SPSS program (version 12.0; SPSS Inc., Chicago, IL, USA) was used. The results were expressed as absolute and relative frequencies, and a descriptive analysis was carried out regarding clinicopathological characteristics. The association between markers and histopathological characteristics was evaluated using the Chi-square (χ2) and Fisher exact tests, and descriptive analysis was carried out. Bonferroni correction was applied to account for multiple tests, adjusting the significance level for the number of comparisons made. Associations with a corrected *p*-value < 0.05 were considered statistically relevant.

## 3. Results

### 3.1. Histopathological Evaluation

Based on the criteria previously mentioned, the classification of the tumors resulted in the following distribution, as shown in Table 2: 10 cases (27.03%) were classified as well-differentiated tumors (Grade I), 15 cases (40.54%) as moderately differentiated tumors (Grade II), and 12 cases (32.43%) as poorly differentiated tumors (Grade III). Additionally, characteristics such as ulceration, necrosis, and vascular emboli were recorded. A majority of the tumors were ulcerated (89.2%), a majority showed areas of necrosis (97.3%), and lymphovascular emboli were observed in seven tumors (18.9%). 

### 3.2. Immunohistochemical Evaluation

#### 3.2.1. Immunohistochemical Expression of EGFR 

**Figure 1:**
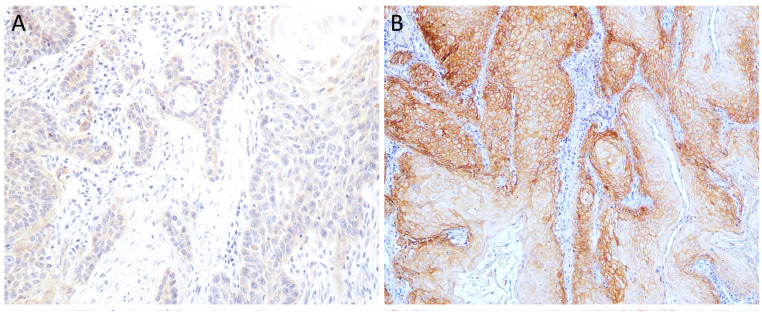
EGFR immunoexpression in tumors with different histological grades of malignancy: (**A**) low score in well-differentiated tumors (Grade I), (100×) and (**B**) EGFR overexpression in poorly differentiated tumors (Grade II), (40×).

EGFR labeling was primarily observed in the cytoplasms and cell membranes of both tumor and normal cells, with the labeling being homogeneous along the cell periphery. Among the tumor cells, 16 samples (43.2%) presented overexpression, while 21 (56.8%) showed low expression, as in Figure 1.

**Figure 2:**
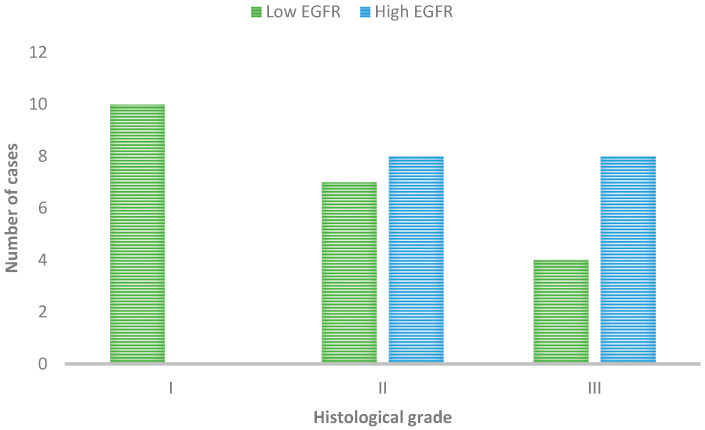
EGFR immunoexpression in tumors with different histological grades of malignancy.

Regarding histopathological parameters, since almost all cases were ulcerated and contained necrotic areas, only histological grade and lymphovascular emboli were considered for statistical purposes. All cases of histological Grade I showed no overexpression of EGFR, as demonstrated in Figure 2. Conversely, most cases in Grades II and III have high EGFR labeling. All of the cases with emboli showed overexpression of EGFR, whereas most cases without did not show overexpression of this receptor (21/30). 

The statistical analysis showed the EGFR expression and the histological grade of malignancy, and the presence of emboli revealed a significant statistical association (*p* = 0.04 and 0.01).

#### 3.2.2. Proliferation Index Detected by Ki-67

**Figure 3:**
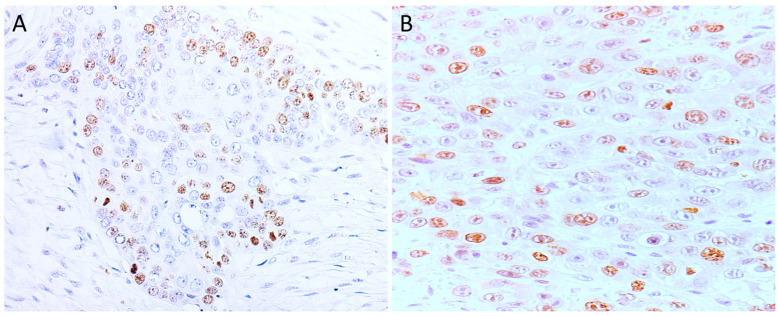
Ki-67 immunoexpression in tumors with different histological grades of malignancy: (**A**) low score in well-differentiated tumors (Grade I), (200×) and (**B**) high score in moderately differentiated tumors (Grade III), (400×).

All cases were positive for Ki-67 in the tumor and in the basal layer of the non-tumoral epidermis adjacent to the tumor. The percentage of positive cells in the analyzed SCCs ranged between 23% and 78%, with a mean of 41.7 and a standard deviation of 9.16. Two categories were created—low and high proliferation—with the cutoff being the mean of positive tumor cells. In total, 18 cases showed low labeling (48.6%), and 19 showed high labeling (51.4%); see Figure 3.

**Figure 4:**
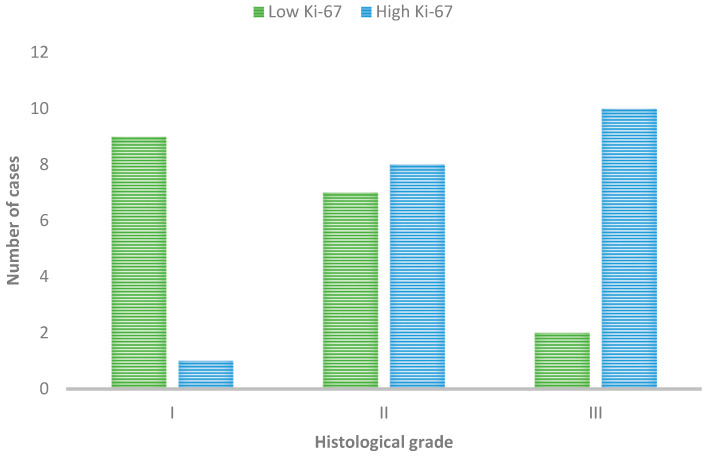
Ki-67 immunoexpression in tumors with different histological grades of malignancy.

Analyzing proliferation and histopathological characteristics, it was observed that all tumors with emboli (7/37) presented high proliferation. Considering the histological grade, most Grade I tumors showed low proliferation, while, in Grade III tumors, the majority (10/12) showed high proliferation, as in Figure 4. The statistical analysis thus revealed a significant association between cell proliferation in SCCs and the histological grade of malignancy, (*p* = 0.02), but not with lymphovascular emboli.

#### 3.2.3. Immunohistochemical Expression of Cox-2

Cox-2 staining was observed predominantly in the cytoplasms of tumor cells. The staining pattern was not uniform across the samples, showing variability in intensity, particularly in areas of invasion where it was more pronounced. 

The staining patterns varied, including dot-like, diffuse granular, or homogeneous distributions within the cytoplasm. Additionally, Cox-2 labeling was detected in macrophages and plasma cells, indicating its presence beyond just tumor cells.

**Figure 5:**
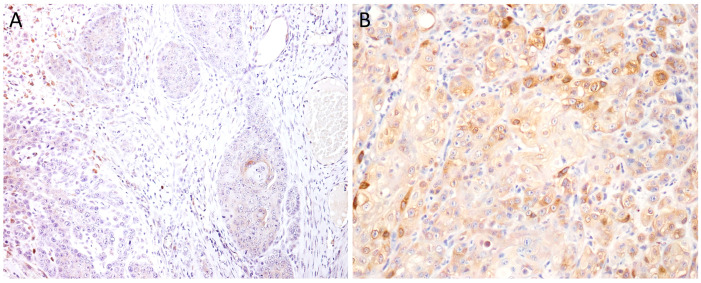
Cox-2 immunoexpression in tumors with different histological grades of malignancy: (**A**) low score in well-differentiated tumors (Grade I), (100×) and (B) high score in poorly differentiated tumors (Grade II), (200×).

A significant majority, 97.3% of cases, exhibited positive staining for Cox-2, with only a single case showing negative staining. Out of 37 cases examined, 16 demonstrated high Cox-2 expression (Figure 5).

Cases with emboli frequently exhibited Cox-2 overexpression, contrasting with most cases without emboli, which showed no such overexpression (20 out of 30 cases). However, no statistically significant association was noted.

**Figure 6:**
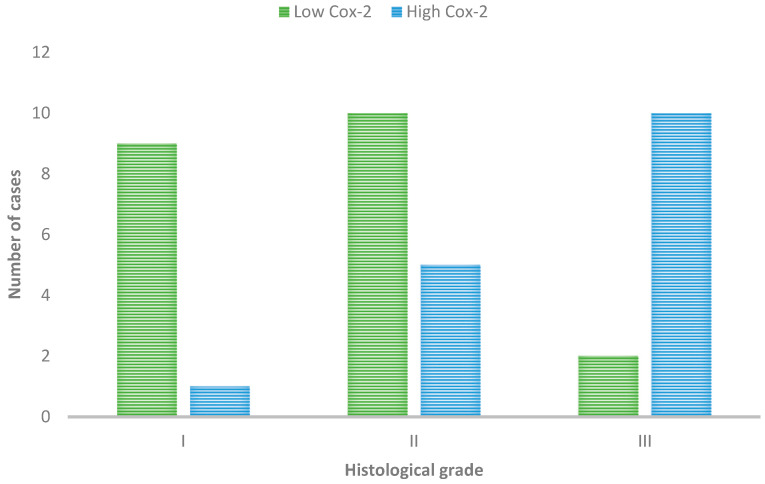
Cox-2 immunoexpression in tumors with different histological grades of malignancy.

Furthermore, Cox-2 expression, categorized into high and low levels, was statistically associated with the histological grade of malignancy (*p* = 0.01). Low Cox-2 expression was predominant in Grade I tumors, with 9 out of 10 tumors showing low levels and only one exhibiting high expression. For Grade II tumors, the majority also displayed low Cox-2 expression (10 out of 15 cases). Conversely, Grade III tumors primarily showed high Cox-2 expression (10 out of 12 cases), suggesting a link between Cox-2 expression levels and tumor grade, as in Figure 6. 

#### 3.2.4. Associations between the Proliferation Index, the Immunoexpression of EGFR, and Cox-2

A statistical association was revealed between Ki-67 and Cox-2 (*p* = 0.02) and between Ki-6 and EGFR (*p* = 0.02). The association between EGFR and Cox-2 was insignificant (*p* = 0.14).

Table 3 reviews the information on the clinical evaluations of histopathological and immunohistochemical findings for each animal included in this study.

## 4. Discussion

Squamous cell carcinoma (SCC) is a significant form of cutaneous neoplasia in dogs, accounting for approximately 4–10% of cases [7]. The molecular changes associated with these carcinomas are not yet fully understood [3,55].

Identifying new tumor markers is crucial in veterinary oncology to better understand and approach these complex cases. So, this study aims to investigate the expression levels of EGFR, Ki-67, and Cox-2 in SCCs by analyzing their associations with the histological grade of malignancy and histopathological features. 

The epidermal growth factor receptor (EGFR) plays key roles in the cell cycle, cell proliferation, cell survival, migration, cell adhesion, and angiogenesis [56,57]. Based on our results, we observed that 43.2% of the tumors studied showed an underexpression of EGFR. These results align with other studies on canine tumors such as oral squamous cell carcinoma [58] and cutaneous squamous cell carcinoma [58]. EGFR overexpression is also commonly observed in various types of human squamous cell carcinomas, such as cutaneous squamous cell carcinoma [59,60], head and neck squamous cell carcinoma [61,62], and oral squamous cell carcinoma [63].

In 43.2% of cases, we noted a consistent overexpression pattern, as previously documented. Furthermore, our investigation unveiled a significant correlation between EGFR expression and tumor malignancy (*p* = 0.04), further substantiating the established connection between EGFR expression levels and tumor aggressiveness. This association has been observed in canine oral squamous cell carcinoma [58], but there are almost no other studies in dogs. Considering human cutaneous squamous cell carcinoma, EGFR overexpression is observed in 70% of cSCCs and is associated with metastasis [51] and poor outcome [59]. However, the association with histological grade is not universal [51]. 

The signaling pathways affected by EGFR activation include RAS-RAF-MEK-MAPK, PLC-gamma/PKC and PI3K/AKT/mTOR, and STAT and NF-kB activation. These pathways play crucial roles in cell proliferation, migration, survival, resistance to apoptosis, and differentiation, all of which are frequently altered in tumors, including squamous cell carcinoma (SCC). Significantly, alterations in the structure or expression of EGFR can trigger tumorigenesis, and the EGFR-STAT3-PD-L1 pathway plays crucial roles in cancer growth and metastasis. Inhibiting this pathway can effectively reduce cancer migration and invasion, suggesting potential therapeutic targets in the treatment of cSCC [64,65,66]. Therapy for cSCC with EGFR inhibitors, including monoclonal antibodies (e.g., cetuximab and panitumumab) and tyrosine kinase inhibitors (gefitinib and erlotinib), has emerged as a promising approach. However, the efficacy of these treatments in human cSCCs is variable [64,65,66]. EGFR inhibitors are better tolerated than chemotherapy, making them a preferred option for human patients who are elderly or have multiple health issues. In such cases, a combined approach using miR-634 ointment and EGFR inhibitors could potentially enhance both the effectiveness and the length of response in treating locally advanced cutaneous squamous cell carcinoma, compared to using EGFR inhibitors alone [67]. Studies in dogs are needed to investigate the effectiveness of these treatments in cutaneous squamous cell carcinomas. 

The Ki-67 protein is a nuclear marker associated with cell proliferation and is present in all cell cycle phases (S, G1, G2, and M) [68]. Its expression occurs in cells that are dividing, but not in quiescent cells (G0), making it a valuable marker for assessing the proliferation rate in various types of cancer [42,45]. The detection of Ki-67 in cancerous tissues helps to characterize the cells’ proliferative activity, providing crucial information for assessing the degree of malignancy and guiding diagnostic and therapeutic strategies [69,70,71].

The results obtained in this study revealed a high expression of Ki-67 in 51.4% of cases. In addition, there was a significant association between Ki-67 expression and the histological grade of malignancy (*p* = 0.02). These findings are consistent with studies in oral canine squamous cell carcinomas (SCCs) [72].

The associations of Ki-67 expression with the histological grade of malignancy reinforce the role of Ki-67 as an indicator of tumor aggressiveness in cases of SCC in dogs. In human cSCC, Ki-67 has not been associated with histological grade [73,74], but with a poor prognosis in cases of laryngeal squamous cell carcinoma [75], head and neck squamous cell carcinoma [76], and oral squamous cell carcinoma [77]. 

A possible future therapy may involve palbociclib, which depletes Ki-67 and cyclin A protein levels, demonstrating its power to inhibit the action of Ki-67 [78]. As Ki-67 measures proliferative activity, any cytotoxic drug that acts on proliferation could be promising for treating tumors with high Ki-67 expression in dogs, which aligns with similar findings in human studies [78,79,80].

Cox-2-mediated prostaglandins, namely PGE2, play a versatile role in various physiological and pathological processes, including blood vessel dilation and increased microvascular permeability [81,82]. The interaction between the different Cox isoforms and the production of prostaglandins, especially PGE2, highlights the central role of these enzymes in regulating the inflammatory response, as well as in various biological processes [83,84].

In our study, 97.3% of the cases were positive for Cox-2, with 16 exhibiting high Cox-2 expression. Previous studies have also shown a high expression levels of Cox-2 in squamous cell carcinoma in dogs in 9 out of 9 [85], 9 out of 9 [86], 10 out of 10 [87], and 7 out of 8 total cases studied [88]. In human tumors, Cox-2 is also expressed in cutaneous SCC [89,90]. 

When comparing Cox-2 expression among tumors of different grades, an association was found between Cox-2 expression and the degree of malignancy (*p* = 0.01). In dogs, as far as we know, no studies compare the expression of Cox-2 across different histological grades in cutaneous SCC. In humans, studies found no correlation between Cox-2 expression in cutaneous SCC and histological grade [91,92]. However, high expression of Cox-2 has been associated with angiogenesis [93], epithelial–mesenchymal transition [94] and prognosis [95]. 

Although Cox-2 expression has been reported in several canine tumors, the therapeutic potential of Cox-2 inhibitors is still subjective, or has not been determined, in most cases [96]. Tumors expressing Cox-2 can be targeted for treatment with Cox-2 inhibitors, such as meloxicam or piroxicam, which have been shown to inhibit cell proliferation and angiogenesis [96,97]. In both human [98,99,100] and veterinary medicine [101,102], targeting Cox-2 with selective Cox-2 inhibitors has been helpful, or could be a promising therapeutic approach for SCC treatment. Prior determination of Cox-2 expression in tumors is crucial for successful treatment with these inhibitors [103,104].

Our results also suggest that prior determination of the immunoexpression of these markers could be useful in clinical practice when evaluating therapeutic options. Given that immunohistochemistry is a relatively easy and accessible method, its routine inclusion in diagnosing these tumors could be crucial. For instance, evaluating Cox-2 expression in tumors may be important for assessing treatment efficacy with Cox-2 inhibitors. Also, using the Ki-67 index to assess tumor cell proliferation could be a helpful tool.

In our study on cutaneous squamous cell carcinomas (cSCCs), we evaluated the expression levels of EGFR, Ki-67, and Cox-2, as well as the possible correlations between them. We observed a significant association between Ki-67 and EGFR (*p* = 0.02), indicating that most cases with EGFR overexpression were associated with high Ki-67 expression. These results are consistent with findings in human brain and breast tumors, where the relationship between EGFR and Ki-67 was also statistically relevant [105]. In the case of human non-small-cell lung cancer, no association has been shown between EGFR and Ki-67 [106] A correlation was observed between EGFR overexpression and a high Ki-67 labeling index, while the opposite was also observed, in that the absence of EGFR expression coincided with a low Ki-67 labeling index. Considering that EGFR is associated with mitosis, cell proliferation, and migration, and Ki-67 is related to proliferative activity, we can infer that an increase in EGFR expression is directly linked to an increase in Ki-67, and vice versa. Similar results were found between Ki-67 and Cox-2 (*p* = 0.02), contrary to those described by Poggiani et al. in 2012, with 10 cases of canine SCC [87], as well as in other tumors, such as human colorectal cancer [107]. Our findings align with those of Escobar et al. (2023), who identified a correlation between less differentiated stages of oral squamous cell carcinomas and increased cellular proliferation rates, thereby facilitating tumor advancement [108]. However, we found no statistically significant associations between EGFR and Cox-2. In contrast, in canine mammary tumors, an association between Cox-2 and EGFR was revealed [103]. Combining various therapies is essential for more satisfactory results [109,110,111], such as an EGFR blocker and a COX-2 inhibitor, as proposed in human advanced SCC [112]. These approaches indicate a promising avenue for treating tumors in dogs, underscoring the necessity for ongoing research to gain deeper insights into the therapeutic advantages they offer.

One limitation of this study is the relatively small number of cases. Several cases initially included in this study had to be excluded, due to technical issues probably related to the fixation and preservation of some samples. However, our results highlight the complexity of molecular interactions in this type of cancer and underline the need for further research with other techniques aside from immunohistochemistry to understand these relationships and their implications for therapeutic approaches.

In summary, molecular research into SCC is crucial to understanding its biology and developing effective strategies in veterinary oncology. Potential tumor markers, such as Cox-2, Ki-67, and EGFR, significantly correlate with tumor characteristics; thus, they could reveal prognosis and, in the cases of Cox-2 and EGFR, therapeutic potential. Future studies may explore specific therapeutic approaches, considering the molecular pathways involved in developing and progressing SCC in dogs.

## 5. Conclusions

These potential tumor markers are significantly associated with the histological grade of malignancy, highlighting their potential as prognostic markers and therapeutic targets in veterinary oncology. 

## Figures and Tables

**Table 1:** Classification of histopathological features [46].

Parameter	Description	Grade I	Grade II	Grade III
Keratinization/Differentiation	The proportion of tumor cells exhibiting keratinization	>50% keratinized cells	20–50% keratinized cells	0–20% keratinized cells
Nuclear Pleomorphism	Maturity of cells	Minimal; >75% mature cells	Moderate; 50–75% mature cells	Marked nuclear pleomorphism
Mitotic Count	Mitoses per ten high-power fields (HPF)	0 to 1 mitosis/HPF	2 to 3 mitoses/HPF	≥4 mitoses/HPF
Invasion Pattern	The pattern of tumor invasion	Well-defined with pushing borders	Infiltration by solid cords, bands, and strands	Infiltration by small groups, strands, or individual cells
Invasion Stage	The extent of tumor invasion	Carcinoma in situ or questionable invasion	Apparent invasion limited to the lamina propria	Invasion beyond the lamina propria involving muscle
Lymphoplasmacytic Infiltration	Level of lymphoplasmacytic infiltration	Marked	Moderate	Mild to absent

**Table 2:** Analyzed data on histological characteristics.

Histological Characteristic	Classification	*n*	%
Ulceration	Absent	4	10.8%
	Present	33	89.2%
Necrosis	Absent	1	2.7%
	Present	36	97.3%
Emboli	Absent	30	81.1%
	Present	7	18.9%
Histological grade of malignancy	I	10	27.03%
	II	15	40.54%
	III	12	32.43%
	Total	37	100%

**Table 3:** Information regarding each animal’s clinical evaluations and histopathological examinations.

Case Number	Breed	Gender	Age	Ulceration	Necrosis	Grade	Embolus	EGFR	Ki-67	Cox-2
1	Indeterminate	female	10	1	1	3	1	1	1	1
2	Boxer	male	8	1	1	2	0	1	0	1
3	Indeterminate	female	11	1	1	1	0	0	1	1
4	Portuguese Pointer	male	11	1	1	2	0	1	0	1
5	Golden Retriever	male	9	1	1	3	1	1	1	2
6	Dalmatian	female	7	1	1	3	0	0	1	1
7	Doberman	female	6	1	1	2	0	1	1	2
8	Estrela Mountain Dog	female	4	1	1	3	1	1	1	2
9	Basset Hound	female	14	1	1	2	0	0	1	2
10	Labrador Retriever	female	7	1	1	2	0	1	1	2
11	West Highland Terrier	male	9	1	1	2	0	0	0	1
12	Dalmatian	male	4	0	0	1	0	0	0	1
13	Portuguese Cattle Dog	male	9	1	1	2	0	1	1	1
14	Dalmatian	male	1	1	1	3	1	1	1	2
15	Podengo	male	11	1	1	3	0	1	0	2
16	Setter	male	9	1	1	2	0	0	0	1
17	Indeterminate	female	8	1	1	3	0	0	1	2
18	Boxer	male	8	1	1	3	0	0	1	2
19	Shar Pei	female	10	1	1	3	1	1	1	2
20	Boxer	male	7	1	1	2	0	0	0	1
21	Pitbull	male	9	0	1	3	1	1	1	2
22	Indeterminate	female	17	1	1	2	0	1	1	1
23	Giant Schnauzer	male	7	1	1	3	1	1	1	2
24	Boxer	male	10	1	1	1	0	0	0	1
25	Boxer	male	9	0	1	1	0	0	0	2
26	Cocker Spaniel	female	10	1	1	1	0	0	0	1
27	Poodle	female	12	1	1	1	0	0	0	1
28	Boxer	female	8	1	1	2	0	1	1	2
29	German Shepherd	male	2	1	1	2	0	1	1	2
30	Boxer	female	9	1	1	1	0	0	0	1
31	Poodle	female	10	1	1	1	0	0	0	1
32	Pekingese	female	6	1	1	2	0	0	1	1
33	Scent hound	female	7	1	1	1	0	0	0	1
34	Boxer	female	7	1	1	2	0	0	0	1
35	Bichon	male	9	0	1	1	0	0	0	1
36	Poodle	female	11	1	1	2	0	0	0	1
37	Partridge	female	7	1	1	3	0	0	0	2

## Data Availability

The data are available upon request from the corresponding author.
